# Micro/Nanocontainer-Based Self-Healing Coatings for Cultural Heritage Conservation

**DOI:** 10.3390/polym18101151

**Published:** 2026-05-08

**Authors:** Wenxuan Chen, Yutong Liu, Shanxiang Xu, Jiaxin Zhang, Xinyou Liu

**Affiliations:** 1 College of Furnishing and Industrial Design, Nanjing Forestry University, Nanjing 210037, China; wenxuanchen@njfu.edu.cn (W.C.); 2311404209@njfu.edu.cn (Y.L.); xushanxiang@njfu.edu.cn (S.X.); 3032751584@njfu.edu.cn (J.Z.); 2 Co-Innovation Center of Efficient Processing and Utilization of Forest Resources, Nanjing Forestry University, Nanjing 210037, China

**Keywords:** corrosion inhibition, cultural heritage, microcapsules, self-healing coating, stimulus-responsive

## Abstract

Micro- and nano-container-based self-healing coatings have emerged as a promising strategy for the long-term conservation of cultural heritage artifacts, including metals, stone, organic matter, and construction materials. These coatings incorporate microcapsules or nanocapsules with tailored shell and core materials, enabling autonomous release of healing agents or corrosion inhibitors in response to damage. For metallic artifacts, benzotriazole@mesoporous silica nanoparticles (*BTA*@*MSN*) microcapsules achieve selective pH-responsive release, reaching 77% at pH 9.0 and 42% at pH 5.0, effectively mitigating localized corrosion. Temperature-adaptive poly(methyl methacrylate-co-methacrylic acid) (*PMMA*-*MA*)/MgO microcapsules exhibit controlled rupture rates, with a 75% reduction at elevated temperatures, enhancing crack repair efficiency by approximately 5%. Organic artifacts, such as wooden or paper manuscripts, benefit from clove oil nanocapsules, which increase tensile strength by 43.5% and fracture toughness by 101.9%, with only 2.91% weight loss over 7 days compared to 33.1% for unencapsulated oil. Advanced fabrication methods—including microfluidics, Pickering emulsions, and multi-core systems—enable high encapsulation efficiency (up to 73.5%), uniform particle size, and repeatable healing. Multi-stimuli responsiveness (pH, temperature, light, magnetic fields) and biobased, environmentally friendly materials further enhance adaptability and sustainability. In this review, “self-healing” is defined broadly to include both physical crack repair and autonomous restoration of protective functions. Overall, self-healing micro/nanocapsule coatings provide a highly controllable, efficient, and durable solution for active heritage protection, representing a shift from passive to intelligent conservation strategies. Furthermore, a systematic comparison of different capsule systems is provided to clarify their respective advantages and limitations. Overall, hybrid systems exhibit the most balanced performance, while inorganic nanocontainers offer superior stability and controlled release, and polymeric capsules enable rapid healing but limited reusability.

## 1. Introduction

Cultural heritage artifacts are invaluable carriers of human history, reflecting the social, artistic, and technological developments of past civilizations. They include not only renowned sculptures and monuments but also countless everyday objects, manuscripts, textiles, and architectural remains that collectively document the trajectory of human creativity and resilience. However, their long-term preservation faces significant challenges due to environmental, chemical, and biological factors. Outdoor bronze sculptures, copper artifacts, and other metallic heritage objects are particularly vulnerable to corrosion, surface oxidation, and patina degradation when exposed to atmospheric pollutants, ultraviolet (*UV*) radiation, and fluctuations in temperature and humidity over extended periods [1,2,3]. Such degradation not only compromises the aesthetic appearance of these artifacts but also threatens their structural integrity. For example, the progressive loss of metal from a bronze statue can lead to the disappearance of fine surface details, such as inscriptions or decorative patterns, which are often of paramount historical significance. Traditional protection methods, including Incralac coatings and benzotriazole (*BTA*) corrosion inhibitors, can slow down deterioration but are limited by the condition of the surface and typically offer protection for only 3–5 years [4,5]. Moreover, organic artifacts, including paper manuscripts, palm-leaf texts, and wooden objects, face additional challenges due to cellulose degradation and the volatilization of naturally active oils, making their long-term stability particularly difficult to guarantee [6]. In many cases, these organic materials are also susceptible to fungal attack, insect infestation, and acidic degradation, which can cause irreversible damage within a relatively short period.

Despite these shared conservation goals, the specific degradation mechanisms and protection requirements vary significantly across different heritage material categories. For metallic artifacts, localized corrosion driven by electrochemical reactions is the primary concern; for stone and masonry, salt crystallization and freeze–thaw cycles dominate; for organic materials (paper, wood, textiles), biological attack and acid-catalyzed hydrolysis are most critical; and for construction materials, microcrack propagation under mechanical and thermal stress is the main issue. Consequently, a universal coating cannot meet all these distinct needs, and the application of self-healing coatings must be tailored to each substrate’s unique deterioration pathway. In particular, most existing studies emphasize the development of coating materials themselves, while the design requirements and substrate-specific compatibility—such as the influence of porosity, mechanical strength, and corrosion sensitivity on microcapsule selection, shell/core types, and fabrication methods—remain largely underexplored. However, current research has largely focused on metallic substrates, leaving a clear research gap in the systematic evaluation of self-healing coatings for other heritage materials.

To overcome the limitations of conventional protective materials, which are often irreversible and have short effective lifespans, micro- and nano-container-based self-healing coatings have recently been introduced in cultural heritage conservation. In line with the conservation principle of minimal intervention, these coatings offer an autonomous, on-demand repair mechanism that reduces the need for repeated manual treatments. This biomimetic approach allows the coating to autonomously respond to damage. Upon the formation of micro-cracks or surface defects, microcapsules or nanocontainers embedded in the coating rupture, releasing healing agents that fill the cracks and repair localized damage [7,8]. This process allows for autonomous partial healing, significantly reducing the need for manual intervention and extending the protective lifespan of the coating. In the context of cultural heritage conservation, the concept of “self-healing” is not limited to the physical repair of cracks or scratches. It also encompasses the autonomous restoration of protective functionality, such as the re-establishment of a corrosion-inhibiting layer or the renewal of an antimicrobial barrier after damage or environmental triggering. Therefore, this review adopts a broader definition that includes both mechanical crack healing (e.g., via polymerizable oils) and functional self-healing (e.g., pH-triggered inhibitor release). Where necessary, the distinction is explicitly noted in the discussion of specific material categories. Early pioneering work demonstrated the feasibility of microcapsule-based self-healing systems, laying the foundation for subsequent developments in the field [9]. Further research has confirmed the effectiveness of these microcapsules on complex surfaces and under low-temperature conditions. Techniques such as fluorescence tracing and electrochemical impedance spectroscopy have been used to verify the localized release of healing agents and the restoration of coating functionality [10]. In addition, recent advances in nanofabrication have enabled the production of microcapsules with precisely controlled size, shell thickness, and release kinetics, thereby expanding their applicability to a wider range of heritage materials, including porous stone, fragile paper, and composite building materials.

**Figure 1:**
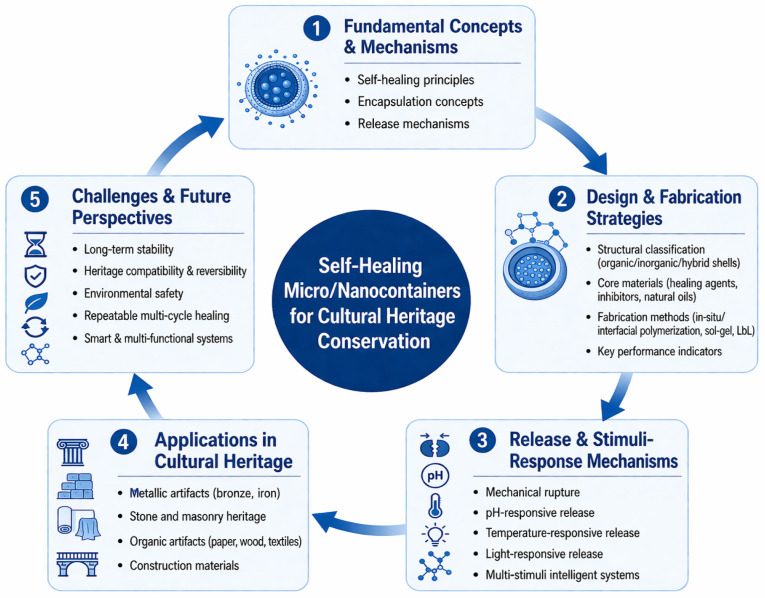
Research framework of the review.

The present review is organized according to a logical framework that progresses from fundamental concepts and mechanisms, through design and fabrication strategies and release and stimuli-response mechanisms, to applications in cultural heritage conservation, before concluding with key challenges and future perspectives (Figure 1).

A dedicated subsection on “Self-healing coatings: Basic Concepts” is provided at the beginning of Section 2 to systematically introduce the definition, classification (organic, inorganic, hybrid shells), core–shell structures, encapsulation and release mechanisms, and various trigger conditions (pH, temperature, light, magnetic field, mechanical stress) along with their typical application scenarios. Initially, the discussion focuses on the design and fabrication of microcapsules, including shell material selection, core agent functionality, and major preparation techniques. Next, the review examines their application across various types of cultural heritage artifacts, including metallic, stone, organic, and building materials. The subsequent section analyzes the mechanisms that trigger the self-healing response, highlighting the roles of pH, temperature, mechanical stress, magnetic fields, and light stimuli. Moreover, existing self-healing systems still face inherent limitations, including insufficient environmental adaptability (e.g., to fluctuating humidity or *UV* radiation), single-use healing capability, and questionable long-term stability under real-world conservation conditions. To systematically evaluate the performance of these coatings, a combination of laboratory characterization methods (e.g., thermogravimetric analysis for thermal stability, nanoindentation for mechanical properties, electrochemical impedance spectroscopy for corrosion resistance) and field-simulated tests (e.g., humidity/temperature cycling, salt spray exposure, *UV* aging) is essential. Furthermore, long-term monitoring indicators—such as crack closure ratio, healing efficiency over multiple damage events, and retention of protective function—are critical for assessing real-world durability. Such performance evaluation not only guides the rational design and optimization of microcapsule-based coatings but also bridges the gap between laboratory research and practical heritage conservation. Following this, the review identifies critical challenges in the field, including reversibility, long-term stability, and dispersion of microcapsules within the coating. Finally, emerging trends in intelligent, green, and repeatable self-healing systems are discussed. By integrating findings from both domestic and international studies, this review aims to provide a comprehensive overview of micro- and nano-container-based self-healing coatings in cultural heritage conservation. The synthesis of current research not only highlights the technical capabilities and limitations of these materials but also offers practical insights for their design, optimization, and application. Ultimately, this work seeks to inform future research and development efforts, providing conservators, material scientists, and engineers with a systematic understanding of self-healing coatings and their potential to revolutionize the preservation of valuable cultural artifacts. This review not only summarizes recent advances in micro/nanocontainer-based self-healing coatings but also provides a systematic comparison of different capsule systems and highlights future research directions toward intelligent and sustainable cultural heritage conservation.

## 2. Fundamental Concepts of Self-Healing Microcapsule Systems

Self-healing coatings have attracted significant attention in recent years as an effective strategy to prolong the service life of materials and reduce maintenance costs [8]. These systems are capable of autonomously repairing damage through the controlled release of healing agents stored within micro- or nanocontainers. When cracks or defects occur in the coating, embedded capsules rupture or respond to external stimuli, releasing active substances that restore the protective function of the coating.

### 2.1. Self-Healing Mechanisms

Self-healing in protective coatings generally relies on the rupture of microcapsules or nanocontainers when cracks propagate through the coating matrix [7,9]. The released healing agents can fill the crack region and form a protective layer through chemical reactions such as polymerization, oxidation, or complexation. For example, drying oils such as linseed oil can undergo oxidative polymerization after exposure to air, forming a crosslinked film that seals the crack. In corrosion protection systems, corrosion inhibitors released from capsules can adsorb onto metal surfaces and suppress electrochemical reactions, thereby slowing corrosion processes.

### 2.2. Encapsulation Strategies

Encapsulation technologies are essential for storing active healing agents within protective shells and ensuring their stability until release is triggered [6]. Various encapsulation strategies have been developed, including in situ polymerization, interfacial polymerization, sol–gel synthesis, and layer-by-layer assembly. Each method produces capsules with different shell structures, sizes, and release characteristics. Polymeric microcapsules prepared via in situ polymerization are widely used due to their simple preparation and high loading capacity, whereas inorganic nanocontainers such as mesoporous silica nanoparticles and layered double hydroxides offer improved chemical stability and controlled release behavior.

### 2.3. Release Mechanisms

The release of healing agents from micro- and nanocontainers can be triggered by several mechanisms depending on the capsule structure and environmental conditions [7,8]. Mechanical rupture is the most common mechanism, in which propagating cracks break the capsule shell and release the encapsulated agent. In addition, stimuli-responsive systems have been developed that respond to environmental triggers such as pH changes, chloride ions, temperature, or light irradiation. These stimuli-responsive systems allow more precise control of release behavior and improve the efficiency of self-healing coatings.

## 3. Design and Fabrication of Microcapsules

Microcapsules serve as the core functional components of self-healing coatings, and their design and fabrication are critical in determining the healing efficiency, functionality, and long-term stability of the coatings. Typically, microcapsules consist of a shell material encapsulating a core agent, which can be a healing agent, corrosion inhibitor, or other functional substance. When micro-cracks or surface damage occur in the coating, the capsules rupture and release their contents, filling defects and restoring local integrity or providing sustained protection. The type of shell and core materials, preparation method, particle size, and encapsulation efficiency all significantly influence the performance and applicability of self-healing coatings [7,11]. A comprehensive comparison of typical micro/nanocapsule systems—including shell materials, core agents, trigger mechanisms, release modes, advantages, limitations, and applications—is provided in Table 1. In recent years, researchers have also explored the possibility of incorporating multiple functional agents within a single microcapsule, such as combining a corrosion inhibitor with a fluorescent self-reporting dye, thereby enabling simultaneous protection and damage monitoring.

### 3.1. Structural Classification

Based on the properties of the shell material, microcapsules can be broadly classified into three categories: organic, inorganic, and organic–inorganic hybrid shells. Organic shells, including polyurethane (*PU*), urea-formaldehyde (*UF*), and polymethyl methacrylate (*PMMA*), exhibit excellent toughness, tunable mechanical properties, and cost-effectiveness, making them widely used in various self-healing systems [12,13,14]. These organic microcapsules can be fabricated with controlled particle size and shell thickness by adjusting reaction conditions, surfactant type, and solvent systems, providing flexibility for tailoring release profiles and mechanical behavior. For instance, by increasing the concentration of the surfactant during emulsion polymerization, typically from 0.5 wt% to 2.0 wt%, one can obtain smaller and more uniformly distributed capsules, which is advantageous for thin coatings where large capsules might cause surface irregularities. Experimental studies have shown that when surfactant concentration is maintained around 1.0–1.5 wt%, the average capsule diameter can be reduced by approximately 30–45%, leading to a more homogeneous dispersion and improving crack-coverage efficiency by up to 20–35%. Overall, polymeric microcapsules provide fast healing and high encapsulation efficiency but suffer from limited reusability and moderate stability.

However, excessively high surfactant concentrations (>2.5 wt%) may reduce encapsulation efficiency (from ~75% down to below 60%) due to interfacial instability, which in turn decreases the effective healing agent availability and slightly lowers the overall healing efficiency. Therefore, an optimal concentration window (approximately 1.0–2.0 wt%) is generally considered to balance capsule uniformity, encapsulation efficiency, and self-healing performance. In contrast, inorganic shells, such as silica (SiO_2_), cerium oxide (CeO_2_), and mesoporous silica nanoparticles (MSNs), offer higher mechanical strength, superior thermal stability, and chemical inertness, which are particularly advantageous for protective coatings on metallic or outdoor heritage artifacts [15,16]. These shells enhance durability under harsh environmental conditions, including *UV* exposure, temperature fluctuations, and moisture. Silica shells, for example, are highly resistant to organic solvents and can protect the core agent from premature degradation, making them suitable for long-term outdoor applications. However, inorganic shells tend to be more brittle than organic ones, and their rupture behavior must be carefully engineered to ensure that they break only when needed. Inorganic nanocontainers exhibit superior thermal and chemical stability as well as controlled release behavior, but their response speed is relatively slow.

Organic–inorganic hybrid shells integrate the flexibility and toughness of organic materials with the robustness and stability of inorganic components, achieving multifunctional performance. Such hybrid capsules can simultaneously provide mechanical self-repair capabilities and corrosion resistance, making them ideal candidates for advanced self-healing coatings in both metallic and non-metallic cultural heritage applications [14,17]. The nanomaterials embedded within the organic matrix reinforce the shell’s mechanical properties and improve thermal stability, ensuring effective protection and prolonged service life. For example, the incorporation of nano-silica into a *PMMA* shell can increase the glass transition temperature and reduce oxygen permeability, thereby extending the shelf life of the encapsulated healing agent. Hybrid systems combine the advantages of both organic and inorganic components, offering balanced performance in terms of healing efficiency, stability, and responsiveness.

**Figure 2:**
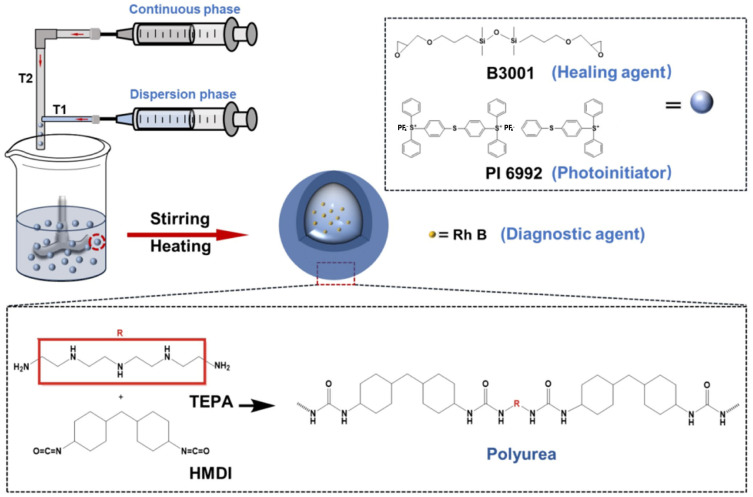
Schematic illustration of the synthesis and composition of polyurea-shell microcapsules for self-healing coatings [17].

Figure 2 illustrates the synthesis and composition of polyurea-shell microcapsules for self-healing coatings. The diagram shows the emulsion-based preparation process, where TEPA and HMDI react to form the polyurea shell, encapsulating a core mixture of healing agent (B3001), photoinitiator (PI 6992), and Rhodamine B (Rh B) as a diagnostic tracer. Polyurea shells provide good mechanical strength and chemical stability, enabling the microcapsules to rupture under mechanical or environmental triggers and release the healing agent on demand. This type of microcapsule design balances structural toughness and functional responsiveness, making it a widely adopted strategy in self-healing coating systems.

### 3.2. Core Materials and Functionality

The selection of core materials directly determines the functional performance of microcapsules in self-healing coatings. In cultural heritage conservation, corrosion inhibitors such as benzotriazole (*BTA*) and 8-hydroxyquinoline (*8*-*HQ*) are commonly employed to respond to early-stage metal corrosion. These agents can be released in response to pH or ion fluctuations at the surface, effectively suppressing further metal degradation [15,18]. *BTA*, for instance, forms a stable complex with copper ions, creating a passivation layer that prevents further oxidation (Cu^2+^ + BTA^−^ → [Cu–BTA]↓ protective coordination complex film). However, *BTA* is toxic, and its long-term environmental impact has raised concerns [19], prompting the search for greener alternatives. In line with green chemistry principles, bio-derived corrosion inhibitors—such as plant polyphenols (e.g., tannic acid), amino acids, and chitosan—have recently been explored as low-toxicity, environmentally friendly substitutes. These natural inhibitors exhibit good compatibility with heritage substrates and can form protective complexes with metal ions, offering a sustainable route for corrosion protection.

Film-forming repair agents, including epoxy resins (EP) and isophorone diisocyanate (*IPDI*), can penetrate microcracks or localized surface damage, restoring the coating’s continuity and providing physical repair [17,20]. These agents typically polymerize upon contact with moisture or atmospheric oxygen, forming a durable film that bridges crack faces. Drying oils such as tung oil, linseed oil, and soybean oil polymerize upon oxidation, forming dense protective films suitable for wooden or paper-based artifacts. Natural active substances, including clove oil and oregano oil, offer additional antimicrobial and antifungal properties, along with sustained release, making them especially useful for organic artifacts exposed to high humidity [21]. The use of these bio-based core materials not only reduces the environmental footprint of self-healing coatings but also aligns with the conservation principles of minimal intervention and reversibility, as they are less likely to cause long-term chemical alterations to the artifacts. In recent studies, mixtures of different oils have been encapsulated to achieve a balance between fast initial release and long-term protection, thereby mimicking the multi-stage healing observed in biological systems.

**Figure 3:**
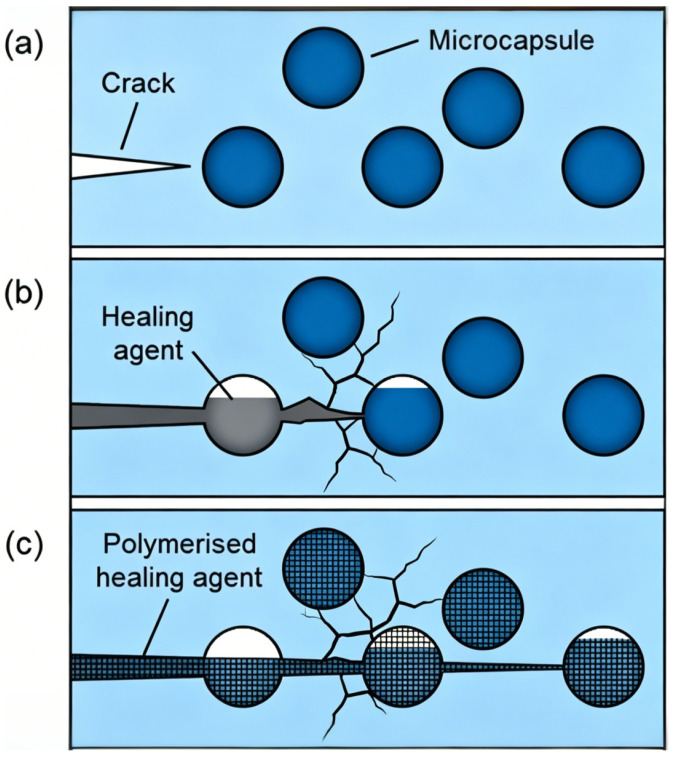
Schematic illustration of the self-healing mechanism of microcapsule-based coatings, showing crack formation, healing agent release, and crack sealing. (**a**) crack formation in the coating matrix, (**b**) rupture of embedded microcapsules and release of the healing agent, and (**c**) polymerization of the healing agent to seal the crack and restore coating integrity.

As illustrated in Figure 3, the basic self-healing mechanism of microcapsule-based coatings follows three main steps: (a) crack formation in the coating matrix, (b) rupture of embedded microcapsules and release of the healing agent, and (c) polymerization of the healing agent to seal the crack and restore coating integrity.

### 3.3. Fabrication Methods

Microcapsules can be fabricated using a variety of techniques, each designed to achieve specific particle sizes, shell thicknesses, and functional requirements [11,22,23]. In situ polymerization involves generating monomers within the oil phase that polymerize at the oil–water interface, yielding microcapsules with high encapsulation efficiency and straightforward operational procedures. This method is particularly suitable for large-scale production because it does not require complex equipment and can be carried out in standard reaction vessels. For example, under temperature-responsive conditions, monomers such as urea–formaldehyde can undergo condensation polymerization to form a crosslinked shell, which can be triggered or accelerated by temperature changes; a simplified representation of the reaction is: n HCHO + n CO(NH_2_)_2_ → (–NH–CH_2_–NH–CO–)ₙ + n H_2_O. In addition, silicate-based microcapsules used in self-healing coatings typically involve the hydrolysis and condensation of siloxane precursors (e.g., TEOS), which can be expressed as: Si(OR)_4_ + 2H_2_O → SiO_2_ + 4ROH, forming a silica-based protective shell. Relevant in situ and temperature-responsive self-healing systems have been reported in previous studies. Interfacial polymerization similarly occurs at the oil–water boundary, producing thin-shelled capsules capable of rapid response to external stimuli. The thickness of the shell can be controlled by adjusting the concentration of monomers and the reaction time, which in turn influences the rupture strength of the capsule [24].

Pickering emulsion polymerization, on the other hand, utilizes solid particles (e.g., silica nanoparticles or clay platelets) to stabilize the emulsion interface without the need for surfactants, allowing the formation of multi-compartment microcapsules suitable for multifunctional coatings. The absence of surfactants reduces the risk of contamination and makes the capsules more biocompatible, which is an advantage when dealing with organic heritage materials that may be sensitive to chemical residues. Microfluidic techniques provide precise control over flow rates and shear forces, enabling the production of highly uniform microcapsules with tightly regulated size distributions, which is particularly advantageous for small-diameter, high-precision applications [17]. Although microfluidics is currently more expensive and less scalable than bulk emulsion methods, it is invaluable for research and development where reproducibility and monodispersity are critical.

Finally, solvent evaporation methods offer a simple laboratory-scale approach, in which the particle size and shell thickness can be modulated by controlling the rate of solvent removal [25]. This method is often used for encapsulating hydrophobic healing agents, but the encapsulation efficiency tends to be lower than that of polymerization-based methods [26]. Collectively, these fabrication strategies provide versatile options for tailoring microcapsule properties to meet the diverse requirements of self-healing coatings in cultural heritage and other advanced material applications [27].

**Figure 4:**
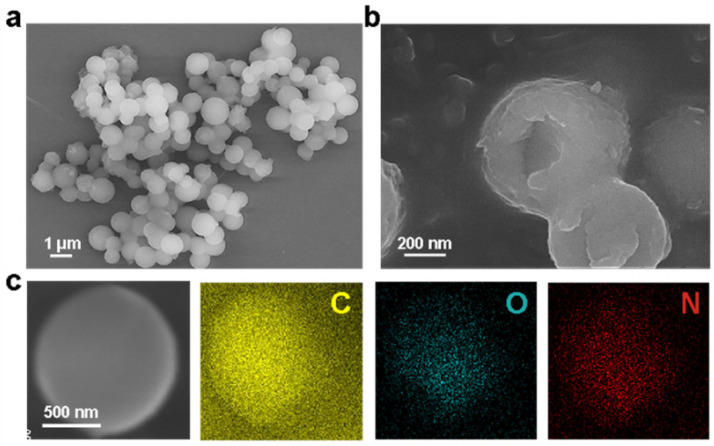
(**a**) *SEM* image of microcapsules. (**b**) SEM image of a broken microcapsule. (**c**) *SEM* image and EDS maps of microcapsules [17].

Figure 4 presents the *SEM* images of microcapsules prepared using microfluidic techniques, showing uniform particle size, smooth shell surfaces, and high encapsulation efficiency. This approach ensures reliable performance and consistent release behavior for self-healing applications.

### 3.4. Key Performance Indicators

The critical performance metrics for microcapsules include thermal stability, mechanical properties, and encapsulation efficiency. Thermal stability is commonly assessed using thermogravimetric analysis (*TGA*) to ensure that microcapsules do not degrade during application or in-service conditions [13,14]. For outdoor heritage conservation, the coating may experience surface temperatures exceeding 60 °C in direct sunlight; thus, the microcapsules must withstand such conditions without premature rupture or core leakage. Mechanical properties are evaluated via nanoindentation or compression testing to confirm that the shell can withstand handling and environmental stress while still rupturing under damage to release the core material [28]. A balance must be struck: if the shell is too strong, it may not break when a crack propagates; if it is too weak, it may rupture during coating application or under normal service conditions.

Encapsulation efficiency affects both the amount of healing agent available for release and the overall self-healing performance, and can be quantified using *TGA* or *UV*–*Vis* spectroscopy [20,29]. High encapsulation efficiency reduces waste and ensures that a sufficient quantity of healing agent is delivered to the damaged site. In practice, encapsulation efficiencies above 70% are considered excellent, but many systems achieve only 40–60%. Researchers are actively developing new emulsification and polymerization protocols to improve this metric [30].

**Figure 5:**
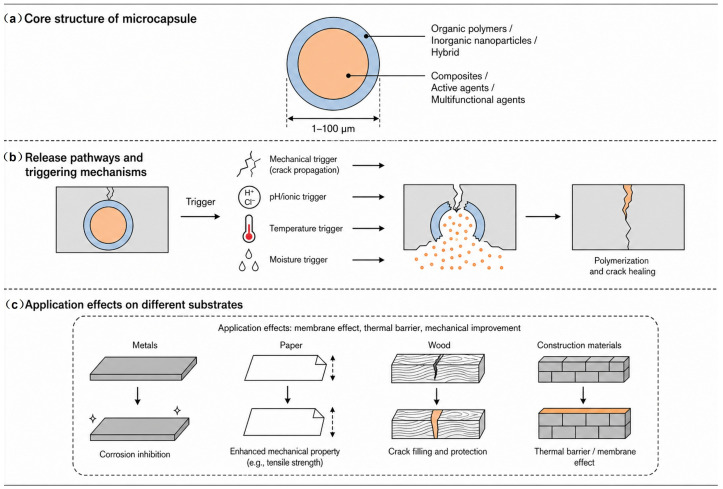
Schematic overview of microcapsule-based self-healing systems for cultural heritage conservation. (**a**) Core structure of microcapsules, showing shell materials (organic polymers, inorganic nanoparticles, or hybrid) and core agents (composites, active agents, or multifunctional agents) with typical size range of 1–100 μm; (**b**) release pathways and triggering mechanisms, including mechanical trigger (crack propagation causing capsule rupture), pH/ionic trigger, temperature trigger, and moisture trigger, followed by polymerization and crack healing; (**c**) application effects on different substrates (metals, paper, wood, and construction materials), highlighting improvements in membrane effect, thermal barrier performance, and mechanical properties.

By carefully optimizing the shell material, core agent, and fabrication method, microcapsules with uniform particle size, high encapsulation efficiency, excellent thermal stability, and robust mechanical performance can be achieved. The overall structure, release pathways, triggering mechanisms, and application effects on different substrates are schematically summarized in Figure 5. These advanced microcapsules provide reliable, long-lasting self-healing functionality, making them highly suitable for the protection of metallic, stone, organic, and building-material artifacts.

### 3.5. Comparative Performance Analysis of Micro/Nanocapsule Systems

**Figure 6:**
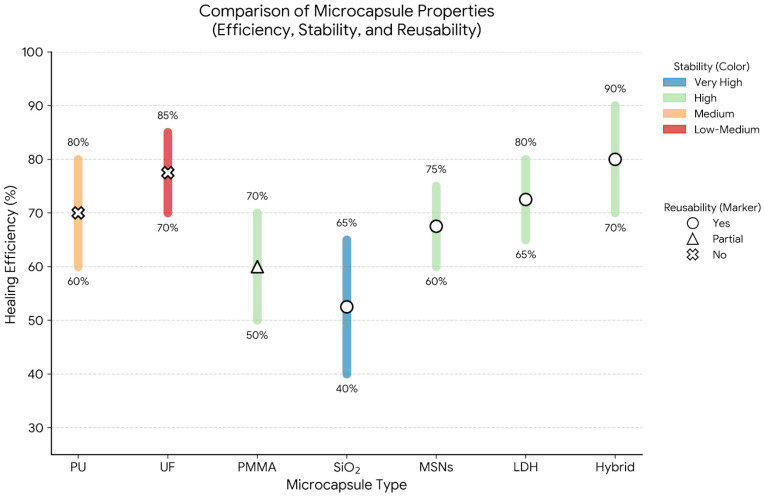
Comparative performance matrix of micro/nanocapsule systems. Values are normalized scores (%), with higher percentages indicating better performance. This matrix visually supports the quantitative comparison in Table 2.

To systematically evaluate the performance differences among various micro/nanocapsule systems, a quantitative comparison is summarized in Table 2. Figure 6 presents a visual performance matrix of these systems. The comparison highlights key parameters, including healing efficiency, release behavior, trigger mechanisms, stability, and reusability, providing a clearer basis for selecting appropriate capsule systems for different cultural heritage applications.

Organic microcapsules, such as polyurethane (*PU*), urea–formaldehyde (*UF*), and polymethyl methacrylate (*PMMA*), generally exhibit relatively high healing efficiencies (typically 60–85%) due to their rapid rupture and efficient release of healing agents upon mechanical damage. However, these systems are predominantly single-use, as the encapsulated agents are depleted after one activation event. In addition, *UF*-based capsules raise concerns regarding toxicity and long-term environmental compatibility, which limit their application in sensitive heritage contexts.

In contrast, inorganic nanocontainers, including silica (SiO_2_), mesoporous silica nanoparticles (*MSNs*), and layered double hydroxides (*LDH*), demonstrate superior chemical and thermal stability, along with controlled and stimuli-responsive release behavior. Although their healing efficiencies (typically 40–75%) may be slightly lower than those of organic systems due to diffusion-controlled release mechanisms, their reusability and long-term durability make them particularly suitable for outdoor and corrosion-prone environments. For example, MSNs and *LDH* systems enable pH- and ion-responsive release, which is highly effective for targeted corrosion inhibition in metallic artifacts.

Hybrid organic–inorganic capsule systems combine the advantages of both categories, achieving high healing efficiency (up to approximately 90%) while maintaining good stability and multi-stimuli responsiveness. These systems support sequential or multi-stage release, enabling repeated or sustained healing processes. As a result, hybrid capsules are considered among the most promising candidates for advanced, intelligent self-healing coatings.

Overall, the selection of micro/nanocapsule systems should be tailored to the specific substrate and degradation mechanism. Organic capsules are more suitable for applications requiring rapid crack sealing, particularly in organic artifacts such as paper and wood. Inorganic nanocontainers are preferred for environments requiring long-term stability and controlled release, such as stone and metallic heritage exposed to harsh conditions.

## 4. Application of Microcapsules in Cultural Heritage Conservation

### 4.1. Metallic Artifacts (Bronzes and Iron Objects)

Metallic artifacts, particularly bronzes, are highly susceptible to corrosion and surface degradation when exposed to outdoor environments over extended periods. For metallic artifacts, the primary self-healing mechanism discussed in this section is functional restoration through the controlled release of corrosion inhibitors or healing agents, rather than physical crack filling. The combined effects of chloride ions, atmospheric pollutants, and fluctuating humidity can lead to the formation of so-called “powdery rust,” resulting in the loss of surface details and structural integrity [31,32]. Traditional protective coatings, such as Incralac and benzotriazole (*BTA*)-based treatments, are limited by short service lifespans and dependency on surface conditions. In particular, *BTA*’s effectiveness varies depending on the type of corrosion products present, and the protective lifespan of such coatings generally ranges from three to five years [33,34]. After this period, the coating must be stripped and reapplied, a process that can be labor-intensive and may cause additional damage to the artifact’s patina.

To overcome these limitations, microcapsule-based self-healing coatings have been introduced into the conservation of metallic artifacts, providing an active, responsive approach to corrosion protection. Table 3 summarizes the main types of micro/nanocontainers used in self-healing coatings for metallic heritage conservation. Several strategies have been developed to optimize the self-healing performance of microcapsules in metallic conservation. pH-responsive release utilizes *BTA* encapsulated in mesoporous silica nanoparticles (*MSNs*), which release the active inhibitor in a controlled manner depending on local pH. For example, under alkaline conditions (pH 9.0), the release rate can reach 77 ± 5%, whereas in acidic conditions (pH 5.0), it decreases to 42 ± 4%, enabling targeted protection in corrosion-prone regions [32]. This selectivity is important because the pH near a corroding metal surface often shifts due to the consumption or production of protons in electrochemical reactions.

**Figure 7:**
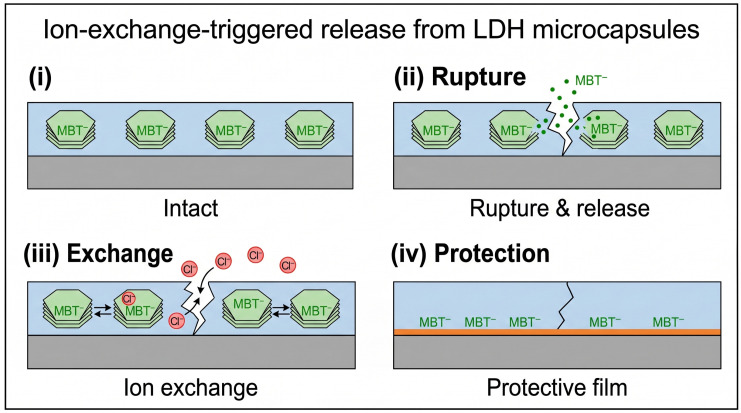
Schematic illustration of the ion-exchange-triggered release mechanism of MBT from *LDH* microcapsules. (**i**) Intact state with MBT loaded; (**ii**) capsule rupture and MBT release; (**iii**) ion exchange process; (**iv**) formation of protective film.

Ion-exchange triggered release employs layered double hydroxide (*LDH*) microcapsules loaded with 2-mercaptobenzothiazole (*MBT*), where changes in environmental ion concentrations induce controlled release, effectively slowing corrosion progression [33]. *LDHs* are anionic clays that can intercalate inhibitor anions; when aggressive ions such as chlorides penetrate the coating, they exchange with the inhibitor, triggering its release. This ion-exchange release process is schematically illustrated in Figure 7. This mechanism provides a direct response to the presence of corrosive species. Magnetically guided targeting leverages magnetic microcapsules directed by an external field to concentrate the healing agents at localized damage sites, increasing local concentration by approximately tenfold while reducing the total required dosage to as low as 0.025 wt% [31]. This approach is particularly promising for treating specific corrosion spots, such as the crevices around rivets or inscriptions, without saturating the entire surface with inhibitor.

Experimental studies have demonstrated that composite microcapsule-based self-healing coatings significantly enhance the corrosion resistance of bronzes, while preserving the stability of the natural patina color and surface aesthetics [32,34]. Moreover, some systems have been shown to withstand accelerated aging tests equivalent to several years of outdoor exposure, indicating that they could offer protection well beyond the three-to-five-year limit of conventional coatings. Recent developments have introduced metal–organic framework (MOF) shells and multi-responsive core–shell designs that offer higher inhibitor loading and more precise pH-triggered release, although long-term stability under ultraviolet exposure and the potential toxicity of novel materials remain challenges that require further investigation.

### 4.2. Stone and Masonry Artifacts

In stone and masonry conservation, self-healing coatings address both physical crack sealing (e.g., via mineral precipitation) and functional protection (e.g., biocide release). The following examples highlight the predominant mechanism in each case. Stone and masonry artifacts are particularly vulnerable to damage caused by microcrack propagation and salt crystallization. Environmental fluctuations, such as temperature changes and humidity variations, can exacerbate these processes, leading to surface flaking, structural weakening, and long-term degradation. Recent advances have highlighted the efficacy of nanocontainer-based systems, particularly mesoporous silica and halloysite nanotubes, for sustained antimicrobial protection and salt damage mitigation in stone substrates [35]. For example, in porous limestone statues, repeated cycles of wetting and drying can cause soluble salts to crystallize within the pores, generating stresses that eventually disintegrate the stone from within. To address these challenges, self-healing coatings based on microcapsules have been developed that respond to temperature or humidity triggers.

For stone conservation, the selection of microcapsules must consider the substrate’s porosity, permeability, and compatibility with original minerals. The primary design principle is to use healing agents that can precipitate within cracks without altering the stone’s natural appearance. In this context, silica nanocontainers loaded with biocides (e.g., 2-mercaptobenzothiazole) have been successfully employed for controlled antifouling release on outdoor stone monuments, ensuring minimal aesthetic impact [36]. Temperature-adaptive microcapsules, such as those with *PMMA*-*MA* shells encapsulating MgO cores, exhibit tunable glass transition temperatures ranging from 20 to 80 °C. At elevated temperatures, although the capsule rupture rate is reduced (by up to 75%) due to shell stiffening, the chemical reactivity of the released MgO is significantly enhanced. This leads to a faster formation of magnesium hydroxide and subsequent carbonate, ultimately improving the crack repair efficiency by approximately 5% compared to ambient conditions [31]. This seemingly counterintuitive behavior—reduced rupture rate but improved healing—can be explained by the fact that at higher temperatures, the released healing agent (MgO) reacts more rapidly with ambient moisture to form magnesium hydroxide, which then carbonates to form a dense filling material(MgO + H_2_O → Mg(OH)_2_; Mg(OH)_2_ + CO_2_ → MgCO_3_ + H_2_O). Sodium silicate microcapsules release their contents into cracks or pores, where they react with cement hydration products to form calcium-silicate-hydrate (*C*-*S*-*H*) gel, providing both mechanical filling and chemical bonding for dual protection [33]. For stone artifacts that are not cementitious, such as marble or sandstone, the reaction with the stone’s natural calcium carbonate can still produce a binding phase, albeit with lower strength.

Over the past three years, several research groups have explored humidity-triggered and biobased microcapsule systems for stone conservation. Notably, MCM-41 mesoporous silica encapsulating plant extracts (e.g., limonene, thyme oil) has emerged as a sustainable alternative, inhibiting fungal growth (*Aspergillus tubingensis*, *Penicillium chrysogenum*) while maintaining stone breathability [37]. Ethyl cellulose shells encapsulating silica precursors have been shown to improve the flexural strength of weathered sandstone, with the advantage of good compatibility with siliceous stone matrices. Calcium alginate-based microcapsules containing calcium hydroxide nanoparticles have been applied to limestone, achieving effective crack sealing with acceptable color matching. The main limitations of these systems include relatively slow healing kinetics under low humidity and the need for careful formulation to avoid surface whitening. Furthermore, hybrid coatings combining nanocontainers with functional nanoparticles (nano-silica, nano-titania) have demonstrated enhanced mechanical strength and *UV* resistance, effectively mitigating salt crystallization and freeze–thaw damage [38]. Additionally, incorporating functional nanoparticles into the microcapsule-based coatings can further enhance the mechanical strength and long-term stability of the coating [32]. For instance, adding nano-silica or nano-titania can improve the coating’s hardness and *UV* resistance, respectively. These strategies effectively mitigate surface deterioration caused by salt crystallization and freeze–thaw cycles, while also exhibiting a degree of environmental self-adaptation, thereby reducing the need for frequent manual restoration.

### 4.3. Organic Artifacts (Paper, Palm-Leaf Manuscripts, and Wood)

For organic artifacts such as paper, wood, and palm-leaf manuscripts, self-healing primarily aims at functional protection (antimicrobial, antifungal) and mechanical reinforcement, with the healing agents typically filling microcracks and forming protective films. The selection of microcapsules is guided by the need for non-toxic, reversible, and visually unobtrusive materials. Natural oils and biobased polymers are preferred over synthetic ones to avoid adverse chemical reactions with the artifact’s original components. Organic artifacts, including ancient manuscripts, textiles, and wooden objects, are prone to cellulose degradation, fungal attack, and structural brittleness over time [31,32]. This degradation is accelerated by environmental stressors, making biobased microcapsules—such as gelatin–arabic gum systems loaded with cinnamon essential oil—promising green solutions for inhibiting mold growth (e.g., Aspergillus niger) on wood and paper [39]. Cellulose, the main structural component of paper and wood, undergoes acid-catalyzed hydrolysis and oxidative cleavage, leading to a progressive loss of mechanical strength. Traditional conservation methods often involve applying synthetic polymers or natural waxes, but these can become yellowed or brittle with age, and they are often irreversible.

Microcapsules loaded with natural oils and essential oils offer an ideal self-healing solution for these materials. Clove oil nanocapsules, encapsulated within *CNW*/*PMMA* shells with particle diameters of approximately 332 nm, shell thickness of 45 nm, encapsulation efficiency of 50.8%, and payload of 35.2%, have been shown to significantly improve mechanical properties when applied to wooden artifacts, with tensile strength increasing by 43.5% and fracture toughness by 101.9%. In a 7-day controlled release test, the weight loss was limited to 2.91%, compared to 33.1% for pure clove oil, demonstrating effective sustained protection [31]. The substantial improvement in fracture toughness is particularly noteworthy, as it indicates that the oil not only lubricates crack faces but also forms a polymerized network (unsaturated oil components undergo oxidative crosslinking: R–CH=CH–R + O_2_ → crosslinked polymeric network) that resists further crack propagation.

Drying oil-based microcapsules, such as tung oil and linseed oil, diffuse slowly into cracks, forming a protective film on the wood surface and providing long-term moisture and microbial resistance [33]. Tung oil, in particular, contains conjugated double bonds that allow it to polymerize rapidly in the presence of oxygen, forming a highly crosslinked film that is both water-repellent and antifungal. For paper conservation, nanocellulose-shelled microcapsules encapsulating thymol or eugenol have been shown to enhance tensile strength while inhibiting fungal colonization, meeting the reversibility and non-toxicity requirements of heritage practice [40]. Moreover, composite microcapsules containing multiple plant oils or active essential oils enable repeated self-healing cycles while maintaining the color and mechanical properties of the artifacts. The morphology and composition of such microcapsules are characterized in Figure 4: low- and high-magnification *SEM* images reveal their spherical shape and uniform shell structure, while elemental mapping confirms the homogeneous distribution of elements consistent with organic shell materials, verifying their suitability for encapsulating natural healing agents.

Recent research on paper and palm-leaf conservation has focused on nanocellulose-based and superhydrophobic microcapsule designs. For example, nanocellulose shells encapsulating essential oils have demonstrated enhanced tensile strength of aged paper along with antifungal activity. Innovative chitosan-based nanocapsules have also been developed for paper deacidification, delivering calcium hydroxide nanoparticles to neutralize acid and stabilize cellulose fibers [41]. For moisture-sensitive palm-leaf manuscripts, microcapsules with wax or stearic acid-modified shells have been proposed to avoid excessive wetting during the healing process. The primary challenges for organic artifact applications include maintaining the original color and texture, ensuring long-term chemical stability of the biobased shells, and preventing any darkening or stiffening of the substrate over time.

### 4.4. Construction Materials

In construction materials like concrete, mortar, and asphalt, self-healing is predominantly physical crack sealing through the reaction of healing agents with the matrix or precipitation of minerals. Self-healing coatings have also been explored for application in construction materials such as cement-based and asphalt-based systems. For concrete and mortar in historical buildings, the self-healing mechanism typically relies on the reaction of healing agents with calcium ions to form carbonate or silicate precipitates that fill cracks. The performance metrics include crack sealing width, recovery of compressive strength, and resistance to carbonation. Recent studies have validated that epoxy- or sodium silicate-loaded microcapsules can restore up to 80% of compressive strength in historic lime mortars, with good compatibility with traditional building materials [42,43]. In cementitious materials, microcapsules release Ca- or Mg-based healing agents during cement hydration, filling microcracks and promoting the formation of cement gel, which restores structural integrity and reduces porosity [32]. For example, calcium nitrate encapsulated in a polymer shell can react with carbonate ions from the atmosphere or from the cement matrix to form calcium carbonate, which effectively seals cracks up to 300 µm in width. This is particularly valuable for historical masonry structures where repointing or grouting would be invasive.

In asphalt-based materials, microcapsules containing drying oils or epoxy resins soften and fill cracks, effectively sealing fissures and delaying aging or material failure [31]. Asphalt pavements on heritage sites, such as old industrial complexes or historic roads, often suffer from thermal cracking and oxidative hardening. The introduction of self-healing microcapsules can extend the service life of such surfaces without altering their historical appearance [44]. Advanced research has further developed dual-capsule systems (epoxy + hardener) for historic mortar repair, achieving high strength recovery while preserving the aesthetic and structural authenticity of heritage masonry [45]. Advanced multifunctional coatings combine microcapsules responsive to pH, temperature, magnetic fields, or light stimuli, enabling targeted repair and repeated self-healing cycles under complex environmental conditions. Such intelligent coatings demonstrate the potential for adaptive protection and autonomous maintenance in large-scale construction materials, extending service life and reducing manual intervention.

Recent advances in construction material self-healing include dual-capsule systems containing epoxy resin and hardener for historic mortar repair, achieving high recovery of compressive strength with good compatibility with lime-based mortars. For earthen architectural heritage (e.g., rammed earth walls), microcapsules incorporating bacterial spores and calcium lactate have been shown to induce biomineralization, sealing microcracks and reducing water infiltration. Notably, bacterial microcapsules (Bacillus cereus) have been optimized for earthen heritage, with calcium carbonate precipitation effectively reducing water permeability by over 50% [46]. The main limitations of these systems are the relatively high cost of microencapsulation and the need for uniform dispersion in thick application layers. Additionally, the long-term durability of bio-based healing agents under varying environmental conditions requires further verification. A comparative overview of microcapsule systems tailored for different cultural heritage materials—including metallic artifacts, stone and masonry, organic artifacts, and construction materials—is provided in Table 4.

## 5. Response Mechanisms

The core principle of micro- and nano-capsule-based self-healing coatings lies in their ability to autonomously repair damage in response to internal or external stimuli. These stimuli can trigger the controlled rupture of capsules, releasing healing agents that restore coating integrity, fill microcracks, and provide localized protection. Release behavior is governed by several physicochemical factors, including shell permeability, shell thickness, diffusion coefficients of the encapsulated agents, and stimulus-induced structural transformations of the capsule wall. These parameters collectively determine the release rate, duration, and ultimate healing efficiency. Depending on the type of stimulus, self-healing coatings are generally categorized into five primary response mechanisms: pH-responsive, temperature-responsive, magnetic-responsive, light-responsive, and mechanically responsive systems [7,47,48]. Each mechanism is designed to address specific environmental or structural challenges, offering tailored protection for different classes of cultural heritage materials.

### 5.1. pH-Responsive Mechanism

pH-responsive coatings are predominantly applied in the conservation of metallic artifacts, where localized corrosion or environmental acid–base fluctuations serve as triggers. Under these conditions, the microcapsule shell undergoes controlled rupture, or structural swelling/degradation, releasing encapsulated corrosion inhibitors such as benzotriazole (*BTA*) or 8-hydroxyquinoline (*8*-*HQ*). These inhibitors then interact with the metal surface to form a protective barrier, effectively mitigating further corrosion (Cu^+^ + BTA → Cu(I)–BTA) [49]. Experimental studies indicate that *BTA*@*MSN* microcapsules release up to 77% of their payload under alkaline conditions (pH 9.0), whereas release is limited to 42% under acidic conditions (pH 5.0), demonstrating both selective sensitivity and targeted functionality [50]. This release follows typical Fickian diffusion kinetics, with an initial burst stage followed by long-term sustained release. This pH-dependent behavior allows the coating to respond dynamically to corrosion-prone zones, providing a highly localized and efficient protective effect. In the context of bronze disease, where localized acidic conditions develop due to the hydrolysis of copper chlorides, pH-responsive capsules could release the inhibitor precisely at the active corrosion front [51].

From a critical perspective, pH-responsive systems offer high selectivity and efficiency (typically 70–80% release within hours) under specific pH shifts, making them ideal for metal corrosion where pH changes are pronounced. However, their limitations include: (i) dependency on the establishment of a sufficient pH gradient, which may be slow in buffered environments; (ii) potential shell degradation over time due to ambient pH fluctuations (e.g., acid rain), leading to premature release; and (iii) the toxicity of many conventional inhibitors (e.g., *BTA*), driving the need for greener alternatives. Therefore, pH-responsive capsules are best suited for indoor or sheltered metal artifacts where pH changes are localized and controlled.

### 5.2. Temperature-Responsive Mechanism

Temperature-responsive microcapsules leverage thermally induced phase transitions to trigger healing. A representative example involves microcapsules with *PMMA*-*MA* shells encapsulating MgO cores, with a tunable glass transition temperature ranging from 20 to 80 °C. When temperatures rise in regions containing microcracks, the shell softens or undergoes a reversible volume transition, the capsules rupture and release healing agents, filling the cracks and restoring the microstructure of the coating [40]. Release kinetics follow an Arrhenius relationship, where higher temperature accelerates molecular diffusion and shell permeability. Similarly, Inozemtcev et al. (2023) [52] investigated capsules containing drying oils [53] and AR polymers in asphalt concrete, showing that elevated temperatures enhance the flow and molecular interpenetration of the healing agents, thereby accelerating the self-repair process. Temperature-responsive systems are particularly useful for stone and masonry materials exposed to seasonal fluctuations or localized heat stress, such as sun-exposed facades or fire-damaged structures.

Critical analysis: Temperature-responsive mechanisms offer the advantage of reversibility (e.g., reversible swelling or melting) and can be designed to trigger at specific temperature thresholds. Healing efficiency can be high (crack recovery > 80%) when the temperature exceeds the glass transition temperature (Tg) or the melting point of the shell material. However, limitations include: (i) the risk of uncontrolled release during hot weather if the trigger temperature is set too low; (ii) the potential for thermal degradation of the healing agent or shell material upon prolonged exposure; and (iii) the requirement for a sufficient temperature rise, which may not occur in consistently cold climates. Consequently, these systems are most effective for outdoor heritage in temperate or sun-exposed locations, or for fire-damaged structures where transient high temperatures occur.

### 5.3. Magnetic-Responsive Mechanism

Magnetic-responsive microcapsules incorporate magnetic nanoparticles (e.g., Fe_3_O_4_) into the core, allowing their positioning and release to be controlled via an external magnetic field. Crall & Keller (2015) [48] demonstrated that magnetic guidance could concentrate capsules at crack sites, increasing the local capsule concentration by up to tenfold. Magnetic trigger induces local shell fracture or enhanced permeability through mild heating or mechanical vibration, enabling precise on-demand release. Remarkably, even when the total capsule loading is as low as 0.025 wt%, efficient healing is achieved. This approach reduces the total amount of healing agent required and minimizes adverse effects on the mechanical properties of the substrate. Magnetic-responsive systems are especially suitable for metallic and composite heritage materials, where targeted release improves both efficiency and precision. For example, a handheld magnet could be used to guide capsules toward a known hairline crack in a bronze statue, releasing the inhibitor exactly where it is needed without coating the entire surface.

Critical analysis: The magnetic mechanism provides unparalleled spatial control and enables on-demand release at specific damage sites, dramatically reducing the required healing agent dosage (as low as 0.025 wt%). Efficiency can be very high (>90% local concentration increase). However, limitations include: (i) the need for an external magnetic field generator, which may not be portable for large or immovable artifacts; (ii) the potential for magnetic nanoparticles to aggregate or cause local heating under alternating fields; and (iii) the requirement that the substrate itself is not strongly magnetic (e.g., iron artifacts may interfere). Therefore, magnetic-responsive capsules are best suited for high-value, localized repairs on non-ferromagnetic metals or composites where precision is paramount.

### 5.4. Light-Responsive Mechanism

Light-responsive microcapsules utilize ultraviolet (UV) or near-infrared (NIR) radiation to initiate chemical reactions that trigger healing agent release and crack repair [17]. Light induces photocleavage, photoisomerization, or photothermal softening of the shell, creating diffusion pathways or direct shell breakdown. The external control of these systems is typically realized by adjusting stimulus intensity, duration, or switching states (on/off), enabling precise regulation of the release behavior. Typically, these systems include photosensitive polymers or photolabile compounds that break down upon exposure to specific wavelengths, releasing the encapsulated agents. Importantly, due to the reversible or re-triggerable nature of certain stimulus–response mechanisms, such systems can exhibit repeatable self-healing capability, allowing multiple damage–healing cycles and significantly improving the long-term durability and service stability of the material. This mechanism is particularly advantageous for non-contact restoration of organic artifacts, such as repairing microcracks in paper manuscripts or wooden surfaces without physical intervention [54]. Light-triggered systems allow precise temporal and spatial control of healing activity, enhancing the protection of delicate or high-value cultural materials. NIR light is especially attractive because it can penetrate deeper into coatings and is less damaging to organic substrates than UV light.

**Figure 8:**
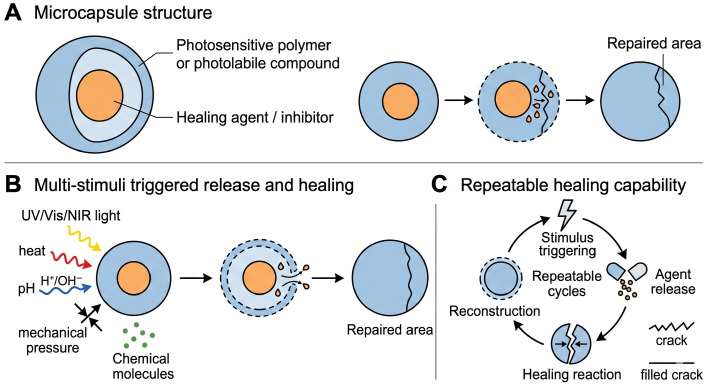
Schematic illustration of microcapsule-based self-healing systems. (**A**) Microcapsule structure showing stimuli-responsive shell (e.g., photosensitive polymer or photolabile compound), encapsulated healing agent, and core/support material; (**B**) multi-stimuli triggered release and healing process under light (*UV*/*Vis*/*NIR*), heat, pH, mechanical pressure, chemical pressure, or specific molecules, leading to shell degradation, healing agent release, and repaired area formation; (**C**) repeatable healing capability over multiple damage cycles, including stimulus triggering, agent release, healing reaction, and reconstruction.

Critical analysis: Light-responsive mechanisms offer exceptional spatial and temporal precision, non-contact operation, and the potential for repeated healing cycles. Healing efficiency can be high (up to 90% recovery) under optimized irradiation conditions. Figure 8 provides a schematic overview of the microcapsule structure, multi-stimuli-triggered release mechanisms, and repeatable healing cycles. However, limitations include: (i) limited penetration depth, especially for UV light, which may not reach deep cracks; (ii) potential photodamage to light-sensitive organic artifacts (e.g., pigments, dyes) if not carefully controlled; (iii) the requirement for specialized light sources (e.g., *NIR* lasers) and long irradiation times; and (iv) the complexity of synthesizing photolabile shell materials. Thus, light-responsive capsules are most suitable for surface-level repairs on organic materials that can tolerate short-wavelength exposure, or for high-precision applications in controlled laboratory settings.

### 5.5. Mechanical-Responsive Mechanism

Mechanical responsiveness constitutes the foundational self-healing mechanism across all microcapsule-based systems. When microcracks propagate through a coating, the local stress causes capsules to rupture via direct shell fracture under stress concentration, releasing healing agents that infiltrate the defect and solidify, effectively restoring coating continuity [55,56]. Release is instantaneous and follows burst-release kinetics, driven by capillary flow and pressure gradients that transport the healing agent into the crack. This mechanism is universally applicable to metals, stones, masonry, and construction materials, providing a versatile and widely effective method for mitigating early-stage damage. Unlike the other mechanisms that require specific environmental conditions, mechanical responsiveness works under virtually any circumstances where a crack forms, making it the most robust and widely used approach.

Critical analysis: The mechanical trigger is the simplest and most reliable, requiring no external stimuli or environmental changes. Its efficiency depends primarily on the mechanical properties of the shell (rupture strength) and the viscosity of the healing agent (capillary flow). Typical healing efficiencies range from 50% to 80% for crack closure. Limitations include: (i) one-time healing per capsule (no repeatability unless multi-core systems are used); (ii) potential for premature rupture during coating application or handling; (iii) difficulty in delivering healing agents to very narrow cracks (<10 µm) due to high capillary resistance; and (iv) the fact that only cracks that intersect capsules are healed. Despite these limitations, mechanical-responsive capsules remain the most practical and widely adopted approach for general heritage conservation, especially when cost and simplicity are priorities.

### 5.6. Multi-Responsive and Intelligent Design

Recent research has focused on multi-stimuli responsive microcapsule coatings, integrating pH, temperature, light, and magnetic triggers within a single system [57]. Such integration enhances sensitivity and functionality, enabling more precise and adaptable self-healing capabilities. In such systems, different stimuli can activate release at different stages of material degradation. For instance, pH changes may trigger early corrosion inhibition, while temperature or light stimuli initiate subsequent healing reactions, providing hierarchical and adaptive protection. These systems achieve synergistic control over shell breakdown and diffusion kinetics, enabling adaptive, sustained, and repeatable release. For example, a capsule could be designed with a shell that is sensitive to both pH and temperature, such that a change in either parameter can induce rupture, providing redundancy and broader applicability. Furthermore, these systems can be combined with additional features, including self-reporting indicators (e.g., color-changing dyes) or antimicrobial functions, broadening the scope of applications in heritage conservation [15,52]. Multi-responsive and intelligent microcapsule coatings represent a significant advancement, allowing cultural artifacts to autonomously adapt to complex environmental conditions while maintaining long-term protection and functional integrity. Overall, each stimulus-responsive mechanism offers distinct advantages depending on environmental conditions and substrate type. Mechanical-responsive systems provide universal applicability, whereas pH- and temperature-responsive systems enable environmentally adaptive release. Magnetic and light-responsive systems allow precise external control. The integration of multiple stimuli-responsive mechanisms is therefore considered a promising strategy for achieving adaptive, repeatable, and long-term protection of cultural heritage materials.

## 6. Challenges and Future Perspectives

### 6.1. Core Challenges

Although micro- and nano-capsule-based self-healing coatings have demonstrated significant advantages in extending the lifespan of cultural heritage artifacts, several technical challenges remain in practical applications. The first major issue is reversibility. In heritage conservation, coatings must often be removable to comply with ethical standards and facilitate future maintenance. However, most current microcapsule-based systems are irreversible; once cured, they are difficult to remove without damaging the underlying substrate [50]. This is particularly problematic for artifacts with sensitive surfaces, such as gilded bronze or polychrome wood, where any mechanical or chemical removal method risks causing additional harm.

The second challenge is long-term stability. The encapsulated healing agents may undergo degradation over time (e.g., polymerization, oxidation, or hydrolysis), and the capsule shell may deteriorate or fail, leading to a gradual loss of self-healing functionality [58,59]. For outdoor heritage exposed to UV radiation, temperature cycling, and pollution, accelerated aging tests have shown that some microcapsules lose up to 50% of their healing capacity after just one year of equivalent exposure. Improving the shelf life of microcapsules without compromising their rupture behavior remains an active area of research.

Thirdly, dispersion remains a critical concern. High concentrations of microcapsules can aggregate, resulting in local clustering that compromises coating uniformity and protective performance [48,60]. Aggregation not only creates weak points in the coating but also reduces the effective surface coverage of healing agents. Even at moderate loadings (5–10 wt%), poor dispersion can lead to large agglomerates that act as stress concentrators, actually promoting crack formation rather than preventing it. Surface modification of microcapsules with compatibilizers or the use of ultrasonic mixing during coating preparation can mitigate this issue, but these approaches add complexity and cost.

Finally, single-use limitations constitute another key bottleneck. Traditional single-core microcapsules can only release their healing agent once; once the capsule ruptures and the healing agent is consumed, the same area cannot be repaired again, leaving the coating vulnerable to subsequent damage events [61]. In real-world scenarios, an artifact may experience multiple cracking events over decades, so a one-time healing capability is often insufficient. This limitation has spurred the development of multi-core or vascular network systems, which are discussed in the next section, as well as multi-stimuli-responsive systems that can achieve repeated healing.

### 6.2. Future Perspectives

**Figure 9:**
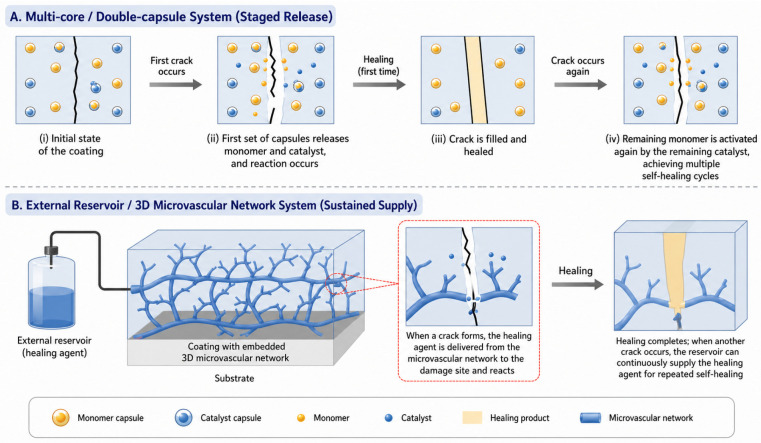
Schematic illustration of repeatable self-healing strategies for microcapsule-based coatings.

To address these challenges, researchers have proposed several strategies. As illustrated in Figure 9, in terms of repeatable self-healing, multi-core or double-capsule systems have been developed to enable staged release of healing agents. Combinations of drying and non-drying oils can form viscoelastic products, allowing multiple healing cycles without the need for external irradiation [60,61]. In some designs, a first set of capsules releases a reactive monomer, while a second set releases a catalyst; after the initial healing, any remaining monomer can be activated again by a later crack event if the catalyst is still present. Another promising approach is the use of three-dimensional microvascular networks embedded in the coating, which can deliver healing agent repeatedly from an external reservoir, although this is more complex to fabricate.

Intelligent coatings utilize multi-stimuli responsiveness—including pH, temperature, light, and magnetic fields—combined with targeted localization to guide microcapsules to areas of concentrated cracks. This approach reduces the total amount of healing agent required while optimizing repair efficiency [48,59]. For example, a coating could be designed so that only the microcapsules near a crack are ruptured by mechanical stress, while those elsewhere remain intact, preserving their healing capability for future damage events. This “on-demand” release is a key step toward truly autonomous and long-lasting protection.

**Figure 10:**
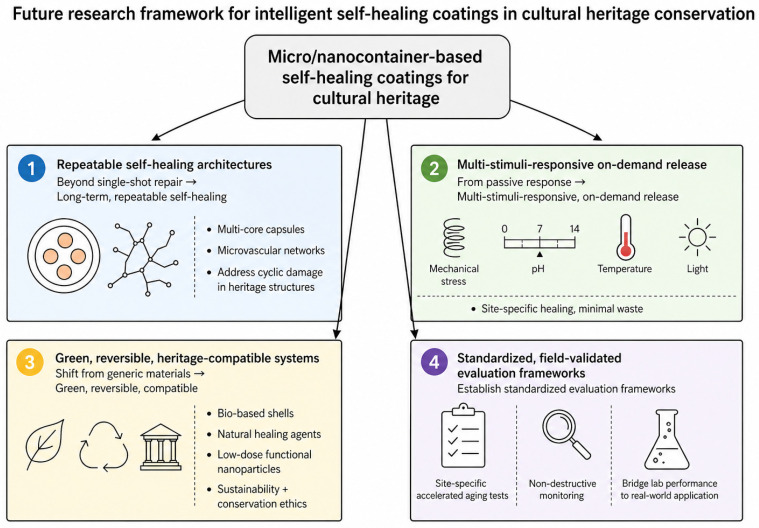
Research framework for future development of intelligent self-healing coatings in cultural heritage conservation.

Looking forward, from the perspective of cultural heritage conservation, future research on micro/nanocontainer-based self-healing coatings should advance along four interconnected, application-driven directions as framed in the research framework presented in Figure 10: moving beyond single-shot repair to develop long-term, repeatable self-healing architectures (multi-core capsules, microvascular networks) that address cyclic damage in heritage structures; evolving from passive response to multi-stimuli-responsive, on-demand release systems tailored to heritage-specific environmental triggers (mechanical stress, pH, temperature, light) to enable site-specific healing while minimizing unnecessary agent consumption; shifting from generic materials to green, reversible, and heritage-compatible systems, combining bio-based shells and natural healing agents with low-dose functional nanoparticles to meet both sustainability and conservation ethics; and establishing standardized, field-validated evaluation frameworks with site-specific accelerated aging tests and non-destructive monitoring to bridge the gap between laboratory performance and real-world heritage applications. These interconnected directions move beyond generic material development to address the unique, practical challenges of cultural heritage conservation, laying the groundwork for translating laboratory breakthroughs into sustainable, long-lasting protection solutions that respect both the material and cultural integrity of heritage assets.

In the green materials domain, biobased shell materials, such as calcium alginate or cellulose, as well as natural plant oils including tung oil, linseed oil, and clove oil, are being extensively explored. These environmentally friendly materials not only enhance sustainability but also improve the degradability and safety of self-healing coatings [52]. For conservators, the use of biobased materials also aligns with the growing preference for “green conservation” approaches that minimize the introduction of synthetic chemicals into the heritage environment. However, the mechanical strength and barrier properties of biobased shells are often inferior to those of synthetic polymers, so further research is needed to optimize their performance.

Moreover, establishing a standardized evaluation system is critical for practical heritage conservation. Most current performance assessments of self-healing coatings rely on controlled laboratory conditions, lacking long-term monitoring methods suitable for complex outdoor or indoor environments. Future research should integrate optical monitoring, microstructural analysis, and electrochemical testing to comprehensively evaluate microcapsule release behavior, healing efficiency, and durability, providing reliable guidance for in situ conservation practices [50,59]. Standardized test protocols—such as scratch-repair tests under controlled humidity, salt spray exposure, and *UV* aging—would allow direct comparison between different microcapsule formulations and facilitate their adoption by conservation professionals.

In summary, micro- and nano-capsule-based self-healing coatings represent a paradigm shift from “passive protection” to “active repair” in heritage conservation. Future development trends are focused on repeatable self-healing, intelligent responsiveness, environmentally friendly materials, and standardized evaluation, providing clear directions for the innovative application of self-healing coatings in cultural heritage preservation.

## 7. Conclusions

Micro- and nano-container-based self-healing coatings have demonstrated significant potential for the long-term protection of cultural heritage materials, offering a transition from passive barrier systems to active, damage-responsive strategies. In particular, polymer-based microcapsules, including polyurethane (*PU*), urea–formaldehyde (*UF*), and polymethyl methacrylate (*PMMA*), remain the most versatile and widely adopted platforms due to their tunable mechanical properties, high encapsulation efficiency, and compatibility with diverse substrates.

This review highlights that the performance of self-healing coatings is strongly dependent on the rational integration of shell materials, core agents, and stimulus-responsive mechanisms, which must be carefully tailored to the degradation characteristics of different heritage materials. Organic capsules enable rapid crack sealing, inorganic nanocontainers provide long-term stability and controlled release, while hybrid systems offer a balanced combination of functionality, durability, and responsiveness. Among these, hybrid polymer–inorganic systems are considered particularly promising for future applications due to their multi-functional adaptability and improved long-term performance.

Despite these advances, several critical challenges remain, including limited reversibility, insufficient long-term stability under real environmental conditions, capsule aggregation, and the single-use nature of conventional microcapsules. Addressing these issues is essential for translating laboratory-scale developments into practical conservation applications.

Future research should therefore focus on the development of multi-responsive and repeatable self-healing systems, particularly those based on advanced polymer architectures, such as multi-core microcapsules and microvascular networks. In addition, the integration of bio-based and environmentally friendly polymer materials is crucial to meet the requirements of sustainability and conservation ethics. Establishing standardized evaluation protocols and long-term field validation methods will also be essential to bridge the gap between experimental research and real-world heritage conservation.

Overall, this review provides a systematic, substrate-oriented design framework for self-healing coatings, offering practical guidance for the selection and optimization of micro/nanocapsule systems in cultural heritage applications. With continued advances in polymer science and intelligent material design, self-healing coatings are expected to play a pivotal role in ensuring the durability, functionality, and sustainability of heritage conservation strategies.

## Figures and Tables

**Table 1:** Performance comparison of different micro/nanocapsule systems used in self-healing coatings, summarizing shell materials, core agents, trigger mechanisms, release modes, key advantages, limitations, and typical application domains.

Shell System	Core Agent	Trigger	Release Mode	Advantages	Limitations	Applications
PU	BTA, drying oils	Mechanical rupture	Burst release	Tough, tunable, scalable	Moderate barrier	Anti-corrosion coatings
UF	Epoxy, linseed oil	Mechanical rupture	Single-shot burst	High encapsulation	Toxicity, brittle	Wood artifact protection
PMMA	Oils, inhibitors	Mechanical/diffusion	Sustained release	Transparent, stable	Lower strength	Heritage coatings
SiO_2_	Epoxy, inhibitors	Mechanical/pH	Controlled diffusion	Thermal and chemical stability	Brittle shell	Stone conservation
CeO_2_	Redox inhibitors	Electrochemical	Autonomous release	Active corrosion protection	High cost	Marine corrosion
MSNs	Oils, inhibitors	pH/ion stimuli	Programmable diffusion	High loading capacity	Brittleness	Smart coatings
PU–SiO_2_	Inhibitors + healers	Mechanical + environment	Sequential release	Tough + barrier synergy	Interface issues	Advanced anti-corrosion
PMMA–SiO_2_	Oils, epoxy	Mechanical/aging	Delayed release	Improved stability	Aggregation risk	Cultural relic protection

**Table 2:** Quantitative performance comparison of typical micro/nanocapsule systems in self-healing coatings for cultural heritage conservation.

Capsule Type	Healing Efficiency (%)	Release Behavior	Trigger Mechanism	Stability	Reusability	Advantages	Limitations	Typical Applications
PU (Polyurethane)	60–80	Fast burst release	Mechanical	Medium	No	Tough shell, scalable, high healing speed	Single-use, moderate durability	Metal coatings, infrastructure
UF (Urea-formaldehyde)	70–85	Instant burst	Mechanical	Low–Medium	No	High encapsulation efficiency, low cost	Toxicity, brittleness	Wood, organic artifacts
PMMA	50–70	Sustained release	Mechanical/thermal	High	Partial	Good transparency, stable release	Lower mechanical strength	Organic heritage (paper, wood)
SiO_2_ (Silica)	40–65	Controlled diffusion	pH/mechanical	Very high	Yes	Excellent chemical stability, durability	Brittle shell, slower response	Stone, outdoor heritage
MSNs (Mesoporous silica)	60–75	Programmable diffusion	pH/ion	High	Yes	High loading capacity, smart release	Structural brittleness	Smart coatings, metals
LDH (Layered double hydroxide)	65–80	Ion-exchange release	Ion-triggered	High	Yes	Self-regulated corrosion protection	Complex synthesis	Metallic artifacts
Hybrid (Organic–Inorganic)	70–90	Multi-stage/sequential	Multi-stimuli	High	Yes	Balanced strength, multifunctionality	Interface compatibility issues	Advanced multifunctional coatings

**Table 3:** Comparison of typical micro/nanocontainers and their performance for cultural heritage protection.

Container Type	Typical Size	Encapsulated Agent	Release Mechanism	Main Advantages	Typical Applications
UF microcapsules	50–200 μm	Drying oils, epoxy	Crack-triggered rupture	High loading; low cost	Metals, wooden artifacts
PU microcapsules	10–100 μm	Epoxy, BTA	Mechanical damage	Tough shell; tunable	Metals, infrastructure
MSNs	50–200 nm	BTA, organic inhibitors	pH/ion diffusion	High stability; controllable release	Bronze, stone relics
LDH nanocontainers	100–500 nm	MBT, vanadate	Ion exchange	Smart release; high corrosion resistance	Metal heritage
Halloysite nanotubes	50–200 nm	Inhibitors, biocides	Diffusion-controlled	Natural; eco-friendly	Stone, construction materials
Polymeric nanocapsules	100–500 nm	Healing monomers	Stimuli-responsive	Multifunctional; compatible	Paper, wood, organic artifacts

**Table 4:** Summary of microcapsule-based self-healing coating systems for different cultural heritage material categories, including degradation mechanisms, recommended microcapsule systems, key advantages, and limitations.

Material Type	Degradation Mechanism	Microcapsule System	Advantages	Limitations
Metallic artifacts	Corrosion, chloride attack, pollutant exposure	pH-responsive MSNs, LDH-based, magnetic microcapsules	Targeted release, high corrosion resistance, patina preservation	UV stability, long-term durability, potential toxicity
Stone and masonry	Salt crystallization, microcracks, freeze–thaw	Temperature/humidity-responsive, silica, MgO, sodium silicate capsules	Crack filling, mineral compatibility, and mechanical strengthening	Slow kinetics in low humidity, whitening risk
Organic artifacts	Cellulose degradation, fungi, oxidation	Natural oil/essential oil capsules, nanocellulose systems	Non-toxic, reversible, improves strength and antifungal properties	Color change risk, long-term stability issues
Construction materials	Cracking, carbonation, thermal aging	Dual-capsules, bacteria-based, epoxy/oil systems	High strength recovery, autonomous repair, large-scale applicability	High cost, dispersion issues, long-term bio stability uncertain

## Data Availability

Data are contained within the article.

## Abbreviations

The following abbreviations are used in this manuscript:

8-HQ	8-Hydroxyquinoline
AFM	Atomic force microscopy
BTA	Benzotriazole
BTA@MSN	Benzotriazole encapsulated in mesoporous silica nanoparticles
CNW	Cellulose nanowhisker
C-S-H	Calcium-silicate-hydrate
EP	Epoxy resin
IPDI	Isophorone diisocyanate
LDH	Layered double hydroxide
MBT	2-Mercaptobenzothiazole
MOF	Metal–organic framework
MSN	Mesoporous silica nanoparticle
NIR	Near-infrared
PMMA	Poly(methyl methacrylate)
PMMA-MA	Poly(methyl methacrylate-co-methacrylic acid)
PU	Polyurethane
SEM	Scanning electron microscopy
TGA	Thermogravimetric analysis
UF	Urea-formaldehyde
UV	Ultraviolet
UV–Vis	Ultraviolet-visible spectroscopy
